# Cingulotomy for Intractable Pain: A Systematic Review of an Underutilized Procedure

**DOI:** 10.7759/cureus.56746

**Published:** 2024-03-22

**Authors:** Billy McBenedict, Wilhelmina N Hauwanga, Mariana P Pires, José Geraldo M Netto, Dulci Petrus, Jumana A Kanchwala, Rhea Joshi, Shaista Rizwan Ahamed Alurkar, Otari Chankseliani, Zaeemah Mansoor, Sona Subash, Berley Alphonse, Ana Abrahão, Bruno Lima Pessôa

**Affiliations:** 1 Neurosurgery, Fluminense Federal University, Niterói, BRA; 2 Family Medicine, Faculty of Medicine, Federal University of the State of Rio de Janeiro, Rio de Janeiro, BRA; 3 Family Health, Directorate of Special Programs, Ministry of Health and Social Services, Namibia, Windhoek, NAM; 4 Medicine and Surgery, Tbilisi State Medical University, Tbilisi, GEO; 5 Faculty of Health Sciences, Karachi Medical & Dental College, Karachi, PAK; 6 Internal Medicine, University Notre Dame of Haiti, Port-au-Prince, HTI; 7 Public Health, Universidade Federal Fluminense, Niterói, BRA

**Keywords:** postoperative pain relief, mri-guided procedures, stereotactic techniques, cancer-related pain, cingulotomy

## Abstract

Pain management is a critical aspect of cancer treatment and palliative care, where pain can significantly impact quality of life. Chronic pain, which affects a significant number of people worldwide, remains a prevalent and challenging symptom for patients. While medications and psychosocial support systems play a role in pain management, surgical and radiological interventions, including cingulotomy, may be necessary for refractory cases. Cingulotomy, a neurosurgical procedure targeting the cingulate gyrus, aims to disrupt neural pathways associated with emotional processing and pain sensation, thereby reducing the affective component of pain. Although cingulotomy has shown promise in providing pain relief, particularly in patients refractory to traditional medical treatment, its use has declined in recent years due to advancements in non-destructive therapies and concerns about long-term efficacy and patient suitability. Modern stereotactic methods have enhanced the precision and safety of cingulotomy, reducing associated complications and mortality rates. Despite these advancements, questions remain regarding its long-term efficacy and suitability for patients with limited life expectancy, particularly those with cancer. A comprehensive systematic review was conducted following the Preferred Reporting Items for Systematic Reviews and Meta-Analyses (PRISMA) 2020 guidelines, aimed at providing insights into the efficacy, potential benefits, and limitations of this neurosurgical procedure in managing intractable pain. An electronic search of PubMed, Embase, Scopus, and Web of Science was conducted with open database coverage dates. The review focused on outcomes such as pain intensity and quality of life. The inclusion criteria encompassed human studies of any age experiencing intractable cancer or non-cancer pain, with cingulotomy as the primary intervention. Various study designs were considered, including observational studies, clinical trials, and reviews focusing on pain and cingulotomy. Exclusion criteria included non-human studies, non-peer-reviewed articles, and studies unrelated to pain or cingulotomy. This review highlights the efficacy of stereotactic anterior cingulotomy in managing intractable pain, particularly when conventional treatments fail. Advanced MRI-guided techniques enhance precision, but challenges like cost and expertise persist. Studies included in this review showed significant pain relief with minimal adverse effects, although the optimal target remains debated. Neurocognitive risks exist, but outcomes are generally favorable. Expected adverse events include transient effects like urinary incontinence and confusion. Reoperation may be necessary for inadequate pain control, with a median pain relief duration of three months to a year. A double stereotactic cingulotomy appears to be safe and effective for refractory pain.

## Introduction and background

Pain and cingulotomy

Pain is the most exasperating symptom among cancer patients, particularly those who suffer from metastatic disease [1]. Hence, the management of pain is a crucial part of treatment and the primary goal of palliative care. The International Association for the Study of Pain (IASP) defines pain as “an unpleasant sensory and emotional experience associated with actual or potential tissue damage or described in terms of such damage” [2]. Pain can be divided into three distinct domains: sensory-discriminative, affective-motivational, and cognitive-evaluative [3]. Chronic pain remains one of the most frequent and disabling symptoms of cancer, impacting mood and potentially leading to feelings of depression, anxiety, and cognitive dysfunction [4]. One of the most prevalent, complicated, and enduring symptoms during and after cancer treatment is pain, which affects about 10 million people worldwide [5]. A recent systematic review, including studies from 2014 to 2021, showed that the overall prevalence of pain in cancer patients was 44% [4]. Studies on pain management, especially in cancer patients, are therefore of great importance.

Several methods of pain management have been designed to overcome this challenge as part of end-of-life care to ease the lives of patients with intractable pain. This includes anxiolytics and pain medications, which mainly include opioids in oral and injectable forms [6]. In addition to medications, social and psychological support systems have also been proven to contribute positively to pain management in cancer patients [6]. However, despite these measures, the pain can become unmanageable over the course of the disease in cancer patients, compelling oncologists to opt for surgical and radiological methods of pain management [7]. In addition, the emerging “opioid crisis” has also warranted the need for interventional therapies for pain management [4]. Alternative interventional therapies like morphine pump, percutaneous neurolysis, stellate ganglion block, cordotomy, cingulotomy, and so on have proven to be more effective in the management of cancer pain refractory to non-interventional therapies [4]. However, patients with widespread pain and those with significant psychological components to their pain may not be appropriate candidates for these interventions [7]. In this situation, cingulotomy has been shown to be effective in treating refractory pain.

Cingulotomy, a neurosurgical procedure, impacts the pain by targeting the cingulate gyrus. The cingulate gyrus plays an important role in processing pain and emotions. It is located in the medial part of the cerebral hemisphere that travels along the corpus callosum and is separated by the pericallosal sulcus [7]. The cingulate gyrus is divided into four subregions: the subgenual and pregenual anterior cingulate cortex, the anterior and posterior midcingulate cortex, the dorsal and ventral posterior cingulate cortex, and the retrosplenial cortex [8]. Classically, the anterior cingulate cortex (ACC) is involved in pain modulation by receiving input from the thalamus and spinal cord [9]. The classical procedure involves creating lesions by radiofrequency within the cingulate white tracts while preserving the frontal U fibers [10].

By disrupting neural pathways related to emotional processing, which produces the sensation of pain [11], cingulotomy aims to reduce the affective component of pain. Destruction of the cingulum bundle and/or cingulate cortex interrupts this connection and, therefore, changes a patient’s perception of pain without changing the sensation of pain per se [11,12]. The “affect” division of the cingulate cortex has been proposed to modulate autonomic activity and internal emotional responses, while the ‘‘cognition” division is involved in responses to noxious stimuli [12]. As a result, postoperatively, patients often experience a decrease in the emotional distress associated with their pain, thereby contributing to an overall improvement in their pain perception and quality of life [13]. The profile of patients that are subjected to cingulotomy is characterized by refractory pain, being non-responsive to medication, seldom being non-neoplastic in nature, diabetic neuropathy, failed back surgery syndrome (FBSS), limb pain following a spinal fracture, a spinal cord injury, and recurrent trigeminal neuralgia [14]. Before surgery, patients would normally exhaust their pharmacological options [11].

History of cingulotomy

Lesioning of the cingulate gyrus, originally used for some psychiatric diseases and then found to alleviate the syndrome of addiction withdrawal, has evolved to be used in the management of patients with medically intractable pain, as it has proven effective in selected patients [12,15]. First introduced by Le Beau in 1954 via open cingulotomy [10], it was inappropriate for terminally ill cancer patients and those incapacitated by chronic pain, general anesthesia, and a major neurosurgical procedure [11]. Later enhanced by Foltz and White in 1962 [10,15], anterior cingulotomy has been successfully performed in the treatment of chronic pain for decades [10,16].

In recent years, the use of anterior cingulotomy has declined in frequency due to a general move away from neuroablative procedures and toward non-destructive procedures such as intrathecal opiate pumps [10,17]. While these newer therapies are reversible, some patients remain refractory, and they are associated with significant costs, perhaps not appropriate for certain populations of patients [10]. For patients with cancer, it is not certain whether the later recurrence of pain results from the late failure of the cingulotomy or the generation of new metastatic foci [11]. Regarding patients with limited life expectancy, Yen et al. considered cingulotomy appropriate [11], in contrast to Sharim and Pouratian who suggested the opposite [10]. Nevertheless, cingulotomy should be considered as an option for patients with malignant pain [16].

Previous stereotactic methods using ventriculography were indirect and cumbersome for lesion localization. The discomfort and complications associated with ventriculography are also well documented. A large lesion was usually necessary to ensure adequate cingulate region coverage by this indirect method [11]. The improvement of stereotactic frames and MRI played a pivotal role in the evolution of stereotactic cingulotomy since their use allowed more accurate targeting and lesioning [17]. Since the introduction of modern stereotactic methods, mortality has rarely been reported [11]. By reviewing the existing literature on cingulotomy and its application in managing intractable pain, the review aimed to provide insights into the efficacy, potential benefits, and limitations of this neurosurgical procedure. This information can be valuable for clinicians and researchers involved in pain management, helping them make informed decisions about the appropriate use of cingulotomy in the context of pain or cancer care.

## Review

Materials and methods

The systematic review utilized the Preferred Reporting Items for Systematic Reviews and Meta-Analyses (PRISMA) principles to guide the compilation, oversight, and reporting of its findings [18].

Source Information and Search Strategy

An electronic literature search across several research databases was conducted, including PubMed, Embase, Scopus, and Web of Science. The search dates were as follows: PubMed was accessed on January 12, 2024; Embase on January 13, 2024; and Scopus and Web of Science on January 14, 2024. The database coverage dates were left open in order to retrieve as much information as possible. Details of the search strategy are provided in Appendix A.

Inclusion and Exclusion Criteria

The inclusion criteria encompassed studies involving humans of any age and experiencing cancer-related intractable pain or non-cancer patients with intractable pain where cingulotomy was the primary intervention for managing pain. Studies with a range of cancer types and stages were considered. The study design included primary research studies and reviews. Outcomes of interest included studies assessing the effectiveness of cingulotomy in alleviating intractable pain, reporting pain intensity, quality of life, and other relevant outcomes. The publication types were limited to peer-reviewed journal articles, and the language was English. Studies that had the following characteristics were excluded: non-human studies, animal studies, or in vitro studies that do not directly inform on cancer and cingulotomy or intractable pain and cingulotomy; non-peer-reviewed articles; conference abstracts; and editorials.

Results

Search Results

**Figure 1:**
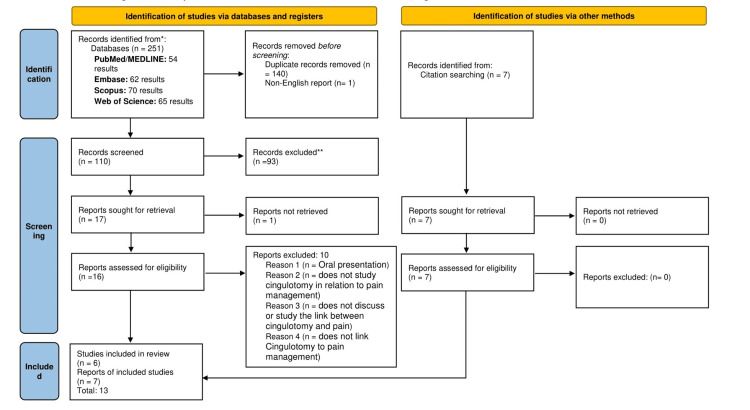
PRISMA flow diagram indicating the steps taken to filter the articles for this review PRISMA, Preferred Reporting Items for Systematic Reviews and Meta-Analyses

Through our search strategy, we identified a total of 251 articles (Figure 1), which included (1) 54 articles from PubMed/MEDLINE; (2) 62 articles through Embase; (3) 70 articles on Scopus; and (4) 65 articles from Web of Science. No filters were applied. The articles were moved to an Excel sheet (Microsoft Corporation, Redmond, Washington, United States), where duplicates and non-English papers were removed manually, leaving us with 110 records. Seventeen remained after removing those with unrelated topics in their title, and these were further reduced to 16 after disqualification based on the abstract. Of these, the full texts of one could not be retrieved, leaving us with 16 papers that we read (full text) for eligibility in accordance with our inclusion/exclusion criteria. Each of the 16 reports that progressed through this screening stage met all the established criteria. Screening of the data was conducted independently by two review authors, with a third reviewer consulted in cases of disagreement. No automated tools were employed in this process. The articles included in the final review were 13 (Table 1).

**Table 1:** Summary of the results of the studies involving patients who underwent cingulotomy CRQ, Chronic Respiratory Questionnaire; MRgLITT, Magnetic Resonance Image-Guided Laser-Induced Thermal Therapy; PIS, Pain Interference Scale; PSS, Patient Satisfaction Scale; VAS, Visual Analog Scale

Author	Patient information	Surgical procedure	Key findings
Sharim and Pouratian (2016) [10]	Mean age: 22 to 85 years; sex: 132 males and 92 females	Not applicable	Across 224 patients in multiple studies, over 60% achieved significant pain relief immediately after surgery, which persisted one year later.
Yen et al. (2005) [11]	Mean age: 35 to 79 years; sex: 13 males and 9 females	Stereotactic bilateral anterior cingulotomy guided by MRI	A total of 12/15 patients with neoplasms experienced meaningful relief at one week, 9/15 at one month, 7/12 survivors at three months, and 5/10 at six months. For patients without cancer, all seven had meaningful relief at one week, 5/7 at one month, 5/7 at three months, and 5/7 at six months and one year.
Yen et al. (2009) [12]	Mean age: 40 to 72 years; sex: 6 males and 4 females	Stereotactic bilateral anterior cingulotomy guided by MRI	One week after surgery, four patients reported experiencing good pain relief, while two reported fair but definite pain relief, and four reported no improvement. These results remained unchanged at the three-month follow-up.
Strauss et al. (2017) [14]	Mean age: 54 ± 14 years; sex: 10 females and 3 males	Standard stereotactic cingulotomy techniques with high-resolution MRI to locate targets	Thirteen patients with oncological pain got immediate pain relief. Three bedridden patients started walking. Pain scores dropped at one-month follow-up, with ongoing relief. Neuropsychological exams showed stable cognitive functions, with occasional confusion or apathy.
Patel et al. (2015) [15]	Mean age: 38 to 51 years; sex: 3 females	Bilateral anterior cingulotomy was performed using laser-induced thermal therapy (MRgLITT) guided by MRI.	A total of 2/3 patients underwent a single MRgLITT procedure, while the third underwent repeat ablation after pain recurrence. The median PSS decreased from 7.7 (range: 7.5-9.3) to 1.6 (range: 1.0-2.8). Furthermore, the median PIS decreased from 9.9 (range: 9.7-10) to 2 (range: 0.3-2.6).
Pereira et al. (2014) [19]	Age: 67 years; sex: males	The anterior cingulotomy procedure was involved.	Opioid use decreased by 80%, and the patients experienced an improvement in pain and dyspnea scores (CRQ and dyspnea subscore VAS).
Wilkinson et al. (1999) [20]	Mean age: 32 to 77 years; sex: 15 males and 8 females	Stereotactic bilateral anterior cingulotomy guided by air ventriculography or CT	One patient had persistent pain for four months before experiencing significant improvement, and two had some relief after reoperation. One patient had excellent pain relief for four years after reoperation, while another had insufficient follow-up.
Pillay and Hassenbusch (1992) [21]	Mean age: 40 to 58 years; sex: 9 males and 3 females	Stereotactic bilateral anterior cingulotomy was performed.	A total of 5/8 patients with neoplasms had good to excellent pain relief. Among patients without cancer, 1/2 experienced relief.
Voris and Whisler (1975) [22]	Not stated	Stereotactic bilateral cingulotomy was performed.	Among patients with neoplasms, 5/5 reported relief from the time of death. Among patients without cancer, 8/11 relief at one to 12 months, 2/11 relief at one year, and 1/11 at three years.
Hurt and Ballantine (1974) [23]	Mean age: 22 to 85 years; sex: 43 males and 25 females	Stereotactic bilateral anterior cingulotomy was used to perform bilateral anterior cingulotomy.	A total of 18/32 patients with neoplasms experienced moderate pain relief within three months, and 2/9 had moderate or complete pain relief within the same timeframe. Moreover, 16/36 patients without cancer achieved complete pain relief in three months or less, and an equal number had moderate to complete pain relief beyond three months.
Faillace et al. (1971) [24]	Mean age: 42 to 66 years; sex: 4 males and 5 females	Stereotactic bilateral anterior cingulotomy was performed using radiofrequency heat ablation.	A total of 3/7 neoplastic patients had pain relief. Moreover, 1/2 non-neoplastic patients had pain relief.
Foltz and White (1968) [25]	Not stated	Unilateral anterior cingulotomy was performed on six patients, and bilateral cingulotomy was conducted on 29 patients.	A total of 9/11 patients with neoplasms experienced good evolution, and 18/24 patients without cancer had a good outcome. Three patients initially had poor pain relief after surgery but achieved good or excellent pain relief after reoperation.
Foltz and White (1962) [26]	Not stated	Unilateral anterior cingulotomy was performed in five patients, and bilateral cingulotomy was conducted in 11 patients.	A total of 5/6 patients with neoplasms experienced a good outcome. Moreover, 6/10 patients without cancer had a good outcome. One patient initially had poor pain relief after surgery for three months but achieved good pain relief after reoperation for four weeks.

Analysis of Study Quality/Bias

The quality of the 11 articles was assessed using the JBI Critical Appraisal Tools (Appendix B). Two articles could not be assessed as sufficient data was not available. The appraisal tool includes questions that focus on the internal validity and risk of bias of study designs, specifically addressing confounding, selection, and information bias, as well as the importance of clear reporting. All studies included in the analysis focused on a clearly defined issue regarding the impact of cingulotomy on pain relief. Each study recruited participants in an acceptable manner, clearly stating both inclusion and exclusion criteria when necessary. However, the criteria were not clearly defined in one study, as indicated in Appendix B. Each study categorized its participants into exposure groups using consistent procedures, which minimizes bias. Various studies utilized different validated scales, such as the Visual Analog Scale (VAS), to precisely measure outcomes and minimize biases. All included studies conducted follow-ups after the procedure, although the duration varied significantly among participants. This discrepancy largely stemmed from some participants passing away shortly after the operation. Such occurrences may introduce bias, as most studies were unable to determine if the outcomes were sustainable. Additionally, many studies exhibited considerable gender disparities among participants, with a notably low number of male participants compared to females. This imbalance makes it challenging to draw meaningful comparisons.

Discussion

Lesions affecting the cingulate gyrus and related structures within the limbic pathway are generally considered interventions of last resort following the unsuccessful application of conventional treatments. Surgical alternatives such as myelotomy and cordotomy are typically reserved for instances of unilateral or visceral midline pain, unaccompanied by significant emotional distress. The integration of image guidance has notably enhanced the minimally invasive nature of cingulotomy procedures [15]. Based on the studies included in this review, the evidence indicates that stereotactic anterior cingulotomy is an effective treatment option. The dorsal ACC is thought to have a major role in cognitive and emotional processing, as well as the perception of pain [27]. Several studies have provided insights into the involvement of the dorsal ACC in modulating the reception of nociceptive information by somatosensory areas [14,28].

Approach to cingulotomy and technological advancement

Modern MRI-guided stereotactic techniques represent a precise and advanced approach to neurosurgical procedures. These techniques utilize MRI to precisely localize and target specific areas within the brain. By using MRI-guided stereotactic techniques, surgeons can navigate and manipulate surgical instruments with exceptional precision, minimizing damage to surrounding healthy tissue and improving patient outcomes. This approach is particularly beneficial in the treatment of brain tumors, movement disorders, epilepsy, and chronic pain conditions. Despite its efficacy, the application of modern MRI-guided stereotactic techniques may be limited by factors such as cost, availability of specialized equipment, and expertise required for interpretation and execution. Modern MRI-guided cingulotomy stereotactic techniques have been primarily reported in small case series [11,12,15,21]. A limited number of eight distinct studies have documented the outcomes associated with stereotactic cingulotomy for the management of cancer-related pain. The reported efficacy of achieving meaningful pain relief among patients ranges from 32% to 83% [7]. Notably, the location of the cingulotomy lesion demonstrates considerable variation, ranging between 1 cm and 4 cm posterior to the tip of the anterior horn. Most case series focused on the placement of a single lesion on each side, positioned approximately 24 mm behind the tip of the frontal horn. However, Strauss et al. incorporated double lesions with the aim of maximizing the potential for long-term efficacy and minimizing the necessity for recurrent operations in vulnerable individuals [14]. This strategic adjustment finds support in studies investigating stereotactic cingulotomies for obsessive-compulsive disorder, where the application of three lesions on each side did not yield an increased risk of neurocognitive adverse effects [29,30]. Although severe adverse events are uncommon, certain transient effects have been observed in the immediate postoperative phase, including diminished focused attention as well as manifestations of apathy and reduced activity levels [7].

Magnetic Resonance Image-Guided Laser-Induced Thermal Therapy (MRgLITT) presently serves as a technique primarily applied in the treatment of traditionally inoperable tumors and epileptic foci [31]. This methodology involves the transmission of laser energy through a stereotactically positioned fiber-optic catheter, demonstrating efficacy in treating tumors within the liver, head-neck region, and intracranial areas [32]. Key attributes of MRgLITT encompass real-time MR guidance and monitoring of thermal ablation. Furthermore, MRgLITT exhibits versatility by accommodating both frame and frameless techniques, utilizing existing navigation systems within the neurosurgeon’s armamentarium. Patel et al. demonstrated the utility of MRgLITT cingulotomy for the treatment of cancer pain refractory to traditional palliative modalities in a series of three patients [15].

Effectiveness of cingulotomy in cancer and non-cancer patients

In a study conducted by Strauss et al., 14 cingulotomy procedures were performed on 13 patients, comprising 10 women and three men [14]. All participants had advanced metastatic cancer with a limited prognosis and were experiencing intractable oncological pain. Following the operation, all patients reported significant pain relief, with no instances of pain exacerbation observed during the study period. Patients experiencing somatic pain from bony metastases typically experience greater pain relief following cingulotomy compared to those with visceral pain. This also tends to have a positive impact on mood and sleep. In addition, 72.7% of the patients reported significant pain relief (>50%), and only 9% reported partial improvement, but in two patients, the pain returned to its original severity [14].

In a study conducted by Sharim and Pouratian, 224 patients underwent cingulotomy procedures [10]. Among these, 149 individuals (67%) experienced significant pain relief postoperatively. Specifically, among the 98 patients with cancer-related pain, 66 (67%) reported significant pain relief, while among the 127 patients with non-neoplastic pain, 83 (65%) had similar relief. Of the total cohort, 156 patients had follow-up data for at least three months, with 87 (56%) experiencing significant pain relief. Among those with a confirmed follow-up of at least three months, 52% of cancer patients (44 out of 84) and 57% of non-neoplastic pain patients (64 out of 112) reported significant pain relief. In addition, palliative bilateral anterior cingulotomy effectively alleviated pain and dyspnea in this terminally ill patient, significantly reducing the distress caused by these symptoms and enhancing their quality of life for two months post-surgery. A total of 94 patients had a follow-up to at least six months post-surgery, of which 59 (63%) were reported to have significant pain relief. Of 20 cancer patients who had follow-ups for at least six months, 12 (60%) had significant pain relief [10]. Likewise, 47 of 74 (64%) patients with non-neoplastic pain with at least six months of follow-up reported significant pain relief. Among 82 patients with at least one year of follow-up, 53 (65%) reported pain relief, including six of nine cancer patients (67%) and 47 of 73 (64%) patients with non-neoplastic pain. The pain VAS recorded its lowest levels four months after the procedure. This is in agreement with Boccard et al.’s findings, in which the pain VAS reached its lowest point four months post-surgery [33].

Hurt and Ballantine reported a series of patients undergoing cingulotomy for persistent pain, and their findings support the effectiveness of the procedure [23]. Among 32 patients with cancer-related pain, 66% experienced some degree of pain relief, while a similar percentage (66%) of the 34 patients with non-neoplastic pain also experienced substantial relief. Wilkinson et al. reported on 18 patients with non-cancer pain, of whom 72% experienced pain improvement [20]. Yen et al. found that 66% of patients with cancer pain reported definite pain improvement at one-month postoperatively, and this proportion decreased to 55% and 50% at three months and six months, respectively [12]. This is in line with the findings of a study that showed that out of 11 patients with intractable oncological pain who were refractory to traditional medical treatment, eight patients exhibited significant pain relief after stereotactic bilateral cingulotomy, whereas one patient experienced partial improvement and two patients returned to their initial state of pain [1].

In Yen et al.’s study of 15 cancer pain patients, 53% reported significant pain relief within the first post-surgery week, 27% experienced meaningful relief, and 20% showed minimal improvement [11]. After one-month follow-up, three patients noted decreased efficacy: one reported less significant relief, and two reverted to pre-surgery pain levels. However, four patients who initially reported slight but meaningful relief still improved. From a total of 12 patients who remained and were evaluated at three months, 33% reported significant relief, 25% experienced meaningful relief, and 42% showed no improvement. Among 10 patients who survived beyond six months, 20% experienced significant relief, 30% had meaningful relief, and 50% showed minimal or no improvement. Among the seven patients with non-cancer pain, two patients with FBSS experienced significant pain relief immediately after cingulotomy. However, one of them reported recurrent pain of the same intensity as before surgery within a few days post-operation, while the others experienced a progressive decline in efficacy, although pain remained moderately relieved during later follow-ups. Another patient with pain following a spinal cord injury initially achieved slight pain relief but reported recurrence at the one-month follow-up. On the other hand, patients with limb pain following spinal cord injury, diabetic neuropathy, and recurrent trigeminal neuralgia responded favorably to cingulotomy, maintaining significant pain improvement at follow-ups of 12, 20, 23, and 12 months, respectively.

Yen et al. conducted stereotactic bilateral anterior cingulotomy procedures aimed at alleviating pain in a cohort of 22 patients with medically refractory pain, comprising 15 individuals with cancer-related pain and seven with non-cancer pain [12]. Among the cancer pain group, 67% of patients reported significant or meaningful pain relief at one-month post-procedure, which declined to 58% at three months and 50% at six months. For patients experiencing non-neoplastic pain, four achieved significant pain relief, one obtained meaningful relief, and two reported no change in pain intensity at the one-year follow-up. In a case series involving three cancer patients, MRgLITT led to successful cingulate gyrus lesions, marked by the absence of adverse surgical reactions and a consistent decrease in symptoms during the postoperative period. Two patients experienced substantial pain reduction, reflected in lower Patient Satisfaction Scale scores, and reported enhanced daily function, as indicated by higher Pain Interference Scale scores. Patients were able to decrease their reliance on pain medication and regain some quality of life [15].

Controversies with the target

Stereotactic anterior cingulotomy has been employed to address refractory oncological or intractable pain by modulating pain perception. Yet, there remains a lack of clear definitions regarding the optimal targets, suitable patient candidates, and outcome measures for this intervention [14]. Controversy regarding the use of cingulotomy remains: first, the mechanism of pain relief is not yet clear. Second, although outcome studies have generally not indicated significant neurocognitive disturbances after cingulotomy, this conclusion is usually derived from subjective clinical observation by clinicians or families. In addition to the well-known ‘‘frontal-type” syndrome involving apathy, decreased activity, and spontaneity, some detailed neurocognitive studies have suggested treatment-related consequences but have generally not documented significant cognitive disturbances [14].

The risk of neurocognitive impairment elicited by cingulotomy remains a potential drawback to the approach. For instance, patients with focal cingulate lesions have demonstrated signs of hemispatial neglect. Ochsner et al. observed deficits in visual cognition and attention in individuals who underwent bilateral cingulotomy [34]. Cohen et al. similarly noted temporary impairments in attention and executive function, along with prolonged deficits in intention and spontaneous response production [13]. Functional imaging studies have further underscored the significance of the ACC in intricate functional brain networks associated with attention and executive control [12].

Commonly observed transient adverse effects encompass urinary incontinence, temporary confusion or disorientation, which subsides within days postoperatively, and mild upper gastrointestinal bleeding, which subsides with medical treatment [11]. Nonetheless, some adverse events may include seizures (<5%), hemiparesis (<1%), personality change (<1%), transient confusion, or mild apathy lasting up to four weeks, which are reported primarily in operations where MR guidance was not used [14]. None of the articles reviewed reported any instances of mortality related to the surgery. However, Yen et al. reported the presence of attentional deficits following surgery using Stroop interference testing [12].

In some cases, some patients are subjected to additional cingulotomy procedures when pain relief is not effective. Sharim and Pouratian reported that some patients required reoperation, specifically a repeat cingulotomy, due to insufficient pain control following the initial surgical intervention [10]. In a total of 224 patients from various studies, a total of 17 reoperations in 16 patients were identified across five reports [15,20,23,25,26]. Among the 17 reoperations, eight resulted in significant pain relief post-surgery. Strauss et al. and Patel et al. also reported the effectiveness of a reoperation cingulotomy performed at an ablation 20 mm anterior to the previous ablation, when the use of additional pain medications failed to control patient symptoms [14,15]. Although cingulotomy is effective for pain relief, the pain may return in some cases. Generally, the median duration of pain relief after surgery is three months to a year [14]. Despite the return of pain in some cases, especially in single-sided cingulotomy, studies showed that double stereotactic cingulotomy is safe and effective in alleviating refractory oncological pain.

Limitations

Some articles included in this review were found to have certain limitations. For instance, in one study, the optimal targets, suitable candidates, and outcome measures were not clearly defined [14]. In all studies, the demographic information could have been more comprehensive, going beyond just age and sex. The shortest follow-up duration ranged from one to 11 months, making it difficult to compare the long-term outcomes of the procedure among participants. In another study, the inclusion and exclusion criteria were not clearly stated, and scales for objectively measuring outcomes were not well defined [22]. The follow-up time for participants was also not clearly indicated. Furthermore, the use of subjective measures such as fair, poor, and excellent for characterizing the study outcome is prone to bias.

## Conclusions

Cingulotomy, particularly when utilizing modern stereotactic techniques guided by advanced imaging modalities such as MRI, presents a promising avenue for addressing refractory oncological pain. The evidence compiled from various studies underscores its effectiveness in providing significant pain relief, not only in cancer patients but also in those with non-neoplastic pain conditions. The integration of image guidance has revolutionized cingulotomy procedures, allowing for precise targeting of the dorsal ACC while minimizing damage to surrounding healthy tissue. Despite the promising outcomes, challenges persist, particularly in defining optimal targets and selecting suitable patient candidates. Moreover, while cingulotomy has generally been associated with manageable transient adverse effects, concerns regarding potential neurocognitive impairments remain. Although studies have not consistently demonstrated significant cognitive disturbances post-procedure, the risk of adverse effects, including attentional deficits and executive function impairments, underscores the need for careful patient selection and ongoing monitoring. Furthermore, the need for reoperations, albeit infrequent, highlights the importance of continued refinement of surgical techniques and comprehensive postoperative care. While cingulotomy offers significant relief for many patients, the potential for pain recurrence, especially in single-sided procedures, necessitates further exploration of strategies to prolong pain relief duration. Overall, despite the remaining challenges and controversies surrounding cingulotomy, particularly regarding target definition and neurocognitive outcomes, the collective evidence suggests that it represents a valuable therapeutic option for patients suffering from intractable oncological pain. Continued research and technological advancements hold the potential to further optimize outcomes and minimize adverse effects, ultimately improving the quality of life for patients experiencing debilitating pain conditions.

## Appendices

Appendix A

**Table 2:** Summary of the search strategy from the databases

Database	Search strategy
PubMed	("cancer pain"[Title/Abstract] OR "malignant pain"[Title/Abstract] OR (("cancer"[Title/Abstract] OR "cancers"[Title/Abstract] OR "malignant neoplasia"[Title/Abstract] OR "malignant neoplasm"[Title/Abstract] OR "malignant neoplastic disease"[Title/Abstract] OR "malignant tumor"[Title/Abstract] OR "malignant tumour"[Title/Abstract] OR "neoplasia, malignant"[Title/Abstract] OR "neoplasmic malignancy"[Title/Abstract] OR "neoplastic malignancy"[Title/Abstract] OR "oncologic malignancy"[Title/Abstract] OR "oncological malignancy"[Title/Abstract] OR "tumor, malignant"[Title/Abstract] OR "tumoral malignancy"[Title/Abstract] OR "tumorous malignancy"[Title/Abstract] OR "tumour, malignant"[Title/Abstract]) AND "pain"[Title/Abstract])) AND ("cingulotomy"[Title/Abstract] OR (("cingulum"[Title/Abstract] OR "cingulum (brain)"[Title/Abstract] OR "cingulum bundle"[Title/Abstract] OR "cingulus"[Title/Abstract] OR "cingular gyrus"[Title/Abstract] OR "cingulate cortex"[Title/Abstract] OR "cingulate gyrus"[Title/Abstract] OR "cortex cinguli"[Title/Abstract] OR "gyrus cinguli"[Title/Abstract]) AND ("ablation therapy"[Title/Abstract] OR "ablation method"[Title/Abstract] OR "ablation methods"[Title/Abstract] OR "ablation procedure"[Title/Abstract] OR "ablation procedures"[Title/Abstract] OR "ablation surgery"[Title/Abstract] OR "ablation technique"[Title/Abstract] OR "ablation techniques"[Title/Abstract] OR "ablation treatment"[Title/Abstract] OR "ablative method"[Title/Abstract] OR "ablative methods"[Title/Abstract] OR "ablative procedure"[Title/Abstract] OR "ablative procedures"[Title/Abstract] OR "ablative surgery"[Title/Abstract] OR "ablative technique"[Title/Abstract] OR "ablative techniques"[Title/Abstract] OR "ablative therapy"[Title/Abstract] OR "ablative treatment"[Title/Abstract] OR "neurosurgery"[Title/Abstract] OR "surgery"[Title/Abstract] OR "method"[Title/Abstract] OR "methods"[Title/Abstract] OR "procedure"[Title/Abstract] OR "procedures"[Title/Abstract] OR "technique"[Title/Abstract])))
Embase	('cancer pain':ti,ab,kw OR 'malignant pain':ti,ab,kw OR (('cancer':ti,ab,kw OR 'cancers':ti,ab,kw OR 'malignant neoplasia':ti,ab,kw OR 'malignant neoplasm':ti,ab,kw OR 'malignant neoplastic disease':ti,ab,kw OR 'malignant tumor':ti,ab,kw OR 'malignant tumour':ti,ab,kw OR 'neoplasia, malignant':ti,ab,kw OR 'neoplasmic malignancy':ti,ab,kw OR 'neoplastic malignancy':ti,ab,kw OR 'oncologic malignancy':ti,ab,kw OR 'oncological malignancy':ti,ab,kw OR 'tumor, malignant':ti,ab,kw OR 'tumoral malignancy':ti,ab,kw OR 'tumorous malignancy':ti,ab,kw OR 'tumour, malignant':ti,ab,kw) AND 'pain':ti,ab,kw)) AND ('cingulotomy':ti,ab,kw OR (((cingulum:ti,ab,kw AND brain:ti,ab,kw OR 'cingulum':ti,ab,kw OR 'cingulum bundle':ti,ab,kw OR 'cingulus':ti,ab,kw OR 'cingular gyrus':ti,ab,kw OR 'cingulate cortex':ti,ab,kw OR 'cingulate gyrus':ti,ab,kw OR 'cortex cinguli':ti,ab,kw OR 'gyrus cinguli':ti,ab,kw) AND ('ablation method':ti,ab,kw OR 'ablation methods':ti,ab,kw OR 'ablation procedure':ti,ab,kw OR 'ablation procedures':ti,ab,kw OR 'ablation surgery':ti,ab,kw OR 'ablation technique':ti,ab,kw OR 'ablation techniques':ti,ab,kw OR 'ablation therapy':ti,ab,kw OR 'ablation treatment':ti,ab,kw OR 'ablative method':ti,ab,kw OR 'ablative methods':ti,ab,kw OR 'ablative procedure':ti,ab,kw OR 'ablative procedures':ti,ab,kw OR 'ablative surgery':ti,ab,kw OR 'ablative technique':ti,ab,kw OR 'ablative techniques':ti,ab,kw OR 'ablative therapy':ti,ab,kw OR 'ablative treatment':ti,ab,kw OR 'neurosurgery':ti,ab,kw OR 'surgery':ti,ab,kw OR 'method':ti,ab,kw OR 'methods':ti,ab,kw OR 'procedure':ti,ab,kw OR 'procedures':ti,ab,kw OR 'technique':ti,ab,kw)))
Scopus	TITLE-ABS-KEY ( ( "cancer pain" OR "malignant pain" OR ( ( "malignant neoplasm" OR "cancer" OR "cancers" OR "malignant neoplasia" OR "malignant neoplasm" OR "malignant neoplastic disease" OR "malignant tumor" OR "malignant tumour" OR "neoplasia, malignant" OR "neoplasmic malignancy" OR "neoplastic malignancy" OR "oncologic malignancy" OR "oncological malignancy" OR "tumor, malignant" OR "tumoral malignancy" OR "tumorous malignancy" OR "tumour, malignant" ) AND "pain" ) ) AND ( "cingulotomy" OR ( ( "cingulum (brain)" OR "cingulum" OR "cingulum (brain)" OR "cingulum bundle" OR "cingulus" OR "cingulate gyrus" OR "cingular gyrus" OR "cingulate cortex" OR "cingulate gyrus" OR "cortex cinguli" OR "gyrus cinguli" ) AND ( "ablation therapy" OR "ablation method" OR "ablation methods" OR "ablation procedure" OR "ablation procedures" OR "ablation surgery" OR "ablation technique" OR "ablation techniques" OR "ablation therapy" OR "ablation treatment" OR "ablative method" OR "ablative methods" OR "ablative procedure" OR "ablative procedures" OR "ablative surgery" OR "ablative technique" OR "ablative techniques" OR "ablative therapy" OR "ablative treatment" OR "neurosurgery" OR "surgery" OR "procedures" OR "method" OR "methods" OR "procedure" OR "procedures" OR "technique" ) ) ) )
Web of Science	TS=(("cancer pain" OR "malignant pain" OR (("malignant neoplasm" OR "cancer" OR "cancers" OR "malignant neoplasia" OR "malignant neoplasm" OR "malignant neoplastic disease" OR "malignant tumor" OR "malignant tumour" OR "neoplasia, malignant" OR "neoplasmic malignancy" OR "neoplastic malignancy" OR "oncologic malignancy" OR "oncological malignancy" OR "tumor, malignant" OR "tumoral malignancy" OR "tumorous malignancy" OR "tumour, malignant") AND "pain")) AND ("cingulotomy" OR (("cingulum (brain)" OR "cingulum" OR "cingulum (brain)" OR "cingulum bundle" OR "cingulus" OR "cingulate gyrus" OR "cingular gyrus" OR "cingulate cortex" OR "cingulate gyrus" OR "cortex cinguli" OR "gyrus cinguli") AND ("ablation therapy" OR "ablation method" OR "ablation methods" OR "ablation procedure" OR "ablation procedures" OR "ablation surgery" OR "ablation technique" OR "ablation techniques" OR "ablation therapy" OR "ablation treatment" OR "ablative method" OR "ablative methods" OR "ablative procedure" OR "ablative procedures" OR "ablative surgery" OR "ablative technique" OR "ablative techniques" OR "ablative therapy" OR "ablative treatment" OR "neurosurgery" OR "surgery" OR "procedures" OR "method" OR "methods" OR "procedure" OR "procedures" OR "technique"))))

Appendix B

**Table 3:** Results for the analysis of study quality or bias using the JBI Critical Appraisal Tools for the included studies

Case Series										
Checklist question	Strauss et al. [14]	Pereira et al. [19]	Voris and Whisler [22]	Pillay and Hassenbusch [21]	Yen et al. [12]	Patel et al. [15]	Foltz and White [26]	Hurt and Ballantine [23]	Yen et al. [11]	Wilkinson et al. [20]
Were there clear criteria for inclusion in the case series?	Yes	Yes	No	Yes	Yes	Yes	Yes	Yes	Yes	No
Was the condition measured in a standard, reliable way for all participants included in the case series?	Yes	Yes	Not clear	Yes	Yes	Yes	Yes	Yes	Yes	Not clear
Were valid methods used for the identification of the condition for all participants included in the case series?	Yes	Yes	Yes	Yes	Yes	Yes	Yes	Yes	Yes	Yes
Did the case series have the consecutive inclusion of participants?	Yes	Yes	Yes	Yes	Yes	Yes	Yes	Yes	Yes	Yes
Did the case series have a complete inclusion of participants?	Yes	Yes	Yes	Yes	Yes	Yes	Yes	Yes	Yes	Yes
Was there clear reporting of the demographics of the participants in the study?	No	Yes	No	No	Yes	Yes	No	Yes	Yes	Yes
Was there clear reporting of clinical information of the participants?	Yes	Yes	Yes	Yes	Yes	Yes	No	Yes	Yes	No
Were the outcomes or follow-up results of cases clearly reported?	Yes	Yes	No	Yes	Yes	Yes	No	Yes	Yes	Yes
Was there clear reporting of the presenting sites’ or clinics’ demographic information?	Not clear	Yes	Not clear	Yes	Yes	Yes	Yes	Yes	Yes	Not clear
Was the statistical analysis adequate?	Yes	Yes	No	No	Yes	Yes	Yes	Yes	Yes	Yes
Systematic Reviews										
Checklist question									Sharim and Pouratian [10]	
1. Is the review question clearly and explicitly stated?									Yes	
2. Were the inclusion criteria appropriate for the review question?									Yes	
3. Was the search strategy appropriate?									Yes	
4. Were the sources and resources used to search for studies adequate?									Yes	
5. Were the criteria for appraising studies appropriate?									Yes	
6. Was critical appraisal conducted by two or more reviewers independently?									Yes	
7. Were there methods to minimize errors in data extraction?									Yes	
8. Were the methods used to combine the studies appropriate?									Yes	
9. Was the likelihood of publication bias assessed?									Yes	
10. Were recommendations for policy and/or practice supported by the reported data?									Yes	
11. Were the specific directives for new research appropriate?									Yes	
